# Depressive symptoms and determinants among people who inject drugs in Kaduna State, Nigeria

**DOI:** 10.4102/phcfm.v16i1.4639

**Published:** 2024-11-08

**Authors:** Nuhu N. Butawa, Awawu G. Nmadu, Sunday Audu, Fidelis O. Ajuonuma, Khadeejah L. Hamza, Sunday Joseph, Abdullateef G. Sule, Joel Adze, Victoria N. Omole, Moroof O. Suleman, Suleiman Usman

**Affiliations:** 1 Department of Prevention Treatment and Care, Kaduna State AIDS Control Agency, Kaduna, Nigeria; 2 Department of Community Medicine, Faculty of Clinical Sciences, College of Medicine, Kaduna State University, Kaduna, Nigeria; 3 Expanded Programme on Immunization (EPI), World Health Organization Country Office, Islamabad, Pakistan; 4 Department of Family Medicine, 44 Nigerian Army Reference Hospital, Kaduna, Nigeria; 5 Department of Community Medicine, College of Medical Sciences, Ahmadu Bello University, Zaria, Nigeria; 6 Department of Planning Research and Statistics, Kaduna State Ministry of Health, Kaduna, Nigeria; 7 Department of Family Medicine, Ahmadu Bello University Teaching Hospital, Zaria, Nigeria; 8 Department of Obstetrics and Gynaecology, College of Medical Sciences, Barau Dikko Teaching Hospital, Kaduna, Nigeria; 9 Department of Obstetrics and Gynaecology, General Hospital Sabon-Tasha, Kaduna State Ministry of Health, Kaduna, Nigeria

**Keywords:** mental health, people who inject drugs, Nigeria, depression, social support

## Abstract

**Background:**

People who inject drugs (PWID) are known to have higher rates of mental health disorders, particularly depression. Despite this, there is a lack of research on the prevalence of depression among PWID in Nigeria.

**Aim:**

This study aimed to determine the prevalence of depressive symptoms and their determinants among PWID.

**Setting:**

The study was conducted in Kaduna State, Northwest Nigeria where a needle and syringe programme (NSP) for PWID was recently initiated.

**Methods:**

A cross-sectional survey was conducted among PWID using a multi-stage sampling technique. Data were collected using structured interviews and digitalised questionnaires covering socio-demographics, depression symptoms (PHQ-9) and perceived social support (MSPSS).

**Results:**

A total of 525 participants were included in the study, with the majority being males (73.3%) aged 24–35 years (54.5%). The study revealed a high prevalence of depressive symptoms among PWID, with 47.4% reporting major symptoms. Predictors of depression identified among PWID included older age, female gender, divorce, unemployment, having primary education and moderate social support.

**Conclusion:**

The study underscored a concerning prevalence of depressive symptoms among PWID, notably linked to diverse sociodemographic factors, emphasising the need for holistic, context-specific mental healthcare approaches for this population.

**Contribution:**

This study contributes to the limited research on the mental health of PWID in Nigeria. It highlights the need for targeted mental health interventions that consider the unique sociodemographic characteristics of this population.

## Introduction

Studies have shown that people who inject drugs (PWID) are more susceptible to a higher prevalence of mental health disorders, notably depression, anxiety and suicidal ideation.^1,2^ A meta-analysis involving 23 studies reported a pooled estimate of 42.0% for current severe depressive symptomatology among PWID.^3^

Previous research identified lower levels of social support as a determinant of depression.^4^ Similarly, a Canadian study discovered that social support was a positive predictor for women who inject drugs and experience depression.^4^ However, no significant statistical association between depression and social support was found, leading them to conclude that social support served a protective role among women experiencing depression.^5^ Other determinants of mental illness in PWID include poverty and malnutrition.^6^ Participants who recently experienced food insecurity had higher than average depression scores.^4^

The unmet needs for mental health treatment among PWID are substantial and often receive low priority. In Kaduna State, there is currently no established policy or plan of action concerning mental health treatment for this population.^7,8,9^ Recognising depressive symptoms early is crucial to enhancing mental health outcomes for PWID. Prioritising the mental health needs of PWID is essential in effectively addressing other potential threats to their overall well-being, such as the global human immunodeficiency virus (HIV) epidemic and other blood-borne infections to which they are particularly prone.^10^

There is a scarcity of studies conducted in Nigeria on the prevalence of mental illnesses and the role of social support in the health outcomes of PWID. To the best of our knowledge, the prevalence of depression among PWID in Kaduna State has not been explored.

Our study aims to determine the prevalence of depressive symptoms and their determinants among people who inject drugs in Kaduna State, Northwest Nigeria.

## Research methods and design

### Study area

The study was conducted in Kaduna State, which is projected to have a population of 10.4 million in 2023 according to the Kaduna State Bureau for Statistics.^11^ This state was selected based on its recent initiation of the Needle and Syringe Programme (NSP), a component of the Harm Reduction Programme. Over 619 PWID have already been enrolled in the programme, and enrolment is ongoing. The Kaduna State Agency for the Control of AIDS (KADSACA) provides these services through drop-in centres situated in Jaji, a community near a military training institution; Ori-apata, a cosmopolitan settlement in Kaduna North Local Government Area; Zaria, with several tertiary institutions including Ahmadu Bello University; General Hospital Sabon Tasha; Sir Patrick Ibrahim General Hospital Kafanchan; and Gwamna Awan General Hospital, Makera Kakuri, which offer secondary services. Other services are provided by non-governmental organisations (Health Action Support Initiative) that focus on community programming.

### Study population and inclusion criteria

The study population included individuals aged 18 years and older who had engaged in drug injection at least once in the month preceding the survey. Eligible participants were not currently enrolled in opioid substitution therapy, as the state was preparing to initiate medicated assisted therapy for opioid-dependent users. Those qualified for participation had provided oral consent after being informed and briefed on the survey details. Excluded from the study were those currently enrolled in opioid substitution therapy.

### Study design

A purposive cross-sectional survey was undertaken among PWID in Kaduna State.

### Sample size

The sample size was determined using the cluster sampling in equation 1:


$$\begin{array}{l} \text{formula}:=DF\frac{Z^{2}\alpha P(1-P)}{e^{2}}\\ DF=1+\rho (m-1) \end{array}$$ [Eqn 1]

Where: P represents the prevalence of severe depression among PWID as reported in a previous study^11^ which was 44%; the level of significance (α) is 5%; *Z*-score corresponding (Z_α_) is 1.96; desired precision (*e*) is 5%; desired sample size is 383; intra-cluster correlation (ρ) is 0.005; the number of individuals in each cluster (*m*) is 50; design effect (DF) is 1.245; required minimum sample size (*n*) is 477; the number of clusters is 5 and after factoring in 10% of non-responses, the sample size increased to 525.

### Sampling method

A multi-stage sampling technique was employed, utilising the Time Location Sampling (TLS) method, a recognised approach for obtaining a probability-based sample from concealed populations.^12^ In the initial stage, Local Government Areas (LGAs) were randomly selected from each of the three geopolitical zones: the northern geopolitical zone with eight LGAs, the central geopolitical zone with seven LGAs and the southern geopolitical zone with eight LGAs. The second stage involved the selection of five high-volume bunks (hotspots – physical locations where injection drug users purchase or obtain their drugs and socialise) in each LGA using a simple random sampling technique from a known list of bunks. The third stage included the random selection and interviewing of available consenting participants based on the inclusion criteria. Interviews were conducted during the participants’ preferred times of drug procurement and usage, specifically between 7:00 and 8:00, and between 16:00 and 18:00.

### Data collection tools and measurement

The questionnaire was administered by interviewers and digitalised using KOBO Collect to enhance the quality of the collected data. The questionnaire encompassed three domains: socio-demographics; Patient Health Questionnaire (PHQ-9) and Multidimensional Scale of Perceived Social Support (MSPSS).

The PHQ-9, a nine-item screening tool, based on criteria for depressive disorders in the Diagnostic and Statistical Manual of Mental Disorders (DSM-IV), was employed to measure symptoms of depression. Respondents were queried about how often, over the past 2 weeks, they had been bothered by specific psychosocial problems associated with depression. Response options were on a four-category Likert scale: not at all (0), several days (1), more than half the days (2) and nearly every day (3). The total scoring ranged from 0 to 27, with a score of ≥ 10 identifying major depression. Scores 0 to 4 indicated no depressive symptoms, while scores equal to or greater than 5, 10, 15 and 20 represented thresholds for mild, moderate, moderately severe and severe depression, respectively. The internal consistency of PHQ-9 was 0.85, with validity (*r* = 0.67), reliability (*r* = 0.894), sensitivity (0.846) and specificity (0.994).^13^

The MSPSS, a 12-item scale, gauged perceived social support from three sources: family, friends and a significant other. Respondents rated their agreement on a 7-point Likert scale ranging from Very Strongly Disagree (1) to Very Strongly Agree (7). Mean scale scores ranging from 1 to 2.9 were considered low support, 3 to 5 denoted moderate support and 5.1 to 7 indicated high support.

### Data collection

In this study, a rigorous data collection process was implemented with a careful selection of research assistants from the PWID community, leveraging their demographic experience. Thorough training equipped them with the skills to collect data responsibly. Communication was standardised in English for consistency. Senior research officers from academia and KADSACA provided diligent supervision, ensuring quality control. Structured interviews lasting approximately 15 minutes per participant were conducted to balance respect for their time with comprehensive data gathering. The 3-month data collection period from June to August 2023 facilitated extensive coverage and inclusion of a diverse participant group.

### Data analysis

The data analysis process in the study involved retrieving data from the Kobo Tool server and importing it into IBM SPSS version 23 for analysis. Initially, univariate analysis explored sociodemographic characteristics, the prevalence of depression and perceived social support by scrutinising each variable individually. Bivariate analysis followed, examining associations between perceived social support and depression symptoms using Chi-square and Fisher’s exact tests. Subsequently, multivariate logistic regression identified predictors of depression. A significance threshold of *P* < 0.05 was set, deeming results below this value statistically significant. Overall, these methods aimed to comprehensively explore relationships and factors influencing depression and social support prevalence.

### Ethical considerations

Ethical clearance to conduct this study was obtained from the Kaduna State Ministry of Health Research and Ethics Committee (No. MOH/ADM/744/VOL.1/111061) before administering the questionnaire. Verbal informed consent was conscientiously acquired from all participants of the study. Rigorous measures were implemented to safeguard the privacy of participants during data collection, and their confidentiality was upheld by gathering anonymous information. Participants were interviewed in settings that ensured their privacy, either at a suitable distance from others or within an enclosure whenever available.

## Results

The age group 25–34 had the highest frequency, comprising 54.5% (286) of the sample, followed by 18–24 at 32.8% (172). Conversely, the age group 35–44 had the lowest frequency and percentage, accounting for only 8.2% (43). Male participants constituted the majority at 73.7% (387), with single individuals making up the largest portion at 65% (341), followed by married individuals at 24.4% (128). Divorced individuals represented the smallest group, comprising only 10.7% (56). In terms of employment status, the private sector had the highest frequency with 33.3% (175), followed by students at 24.6% (129) and the public sector at 20.8% (109). Unemployed individuals accounted for 21.3% (112). Regarding the level of education, the tertiary education group had the highest frequency and percentage at 48.4% (254), followed by secondary education at 31.8% (167) and primary education at 3.8% (20). The combination of no education and primary education accounted for 20.8% (104). In terms of the type of drugs injected, ‘Injection (Not specified)’ had the second-highest frequency at 35.2% (185), while Pentazocine was the most frequent at 60.4% (317). Other drug types had lower percentages, ranging from 0.2% to 2.9% (Table 1).

**TABLE 1:** Frequency and percentage of demographic and drug use characteristics among people who inject drugs in Kaduna State (*N* = 525).

Variable	Frequency	%
**Age**		
18–24	172	32.8
25–34	286	54.4
35–44	43	8.2
≥ 45	24	4.6
**Sex**		
Female	138	26.3
Male	387	73.7
**Marital status**		
Single	341	64.9
Married	128	24.4
Divorced	56	10.7
**Employment status**		
Student	129	24.6
Employed (private sector)	175	33.3
Employed (public sector)	109	20.8
Unemployed	112	21.3
**Level of education**		
None	84	16.0
Primary	20	3.8
Secondary	167	31.8
Tertiary	254	48.4
**Type of drugs injected**		
Amphetamine	15	2.9
Codeine	3	0.6
Flunitrazepam	2	0.4
Injection (not specified)	185	35.2
Morphine	1	0.2
Nitrazepam	2	0.4
Pentazocine	317	60.3

Nearly half of the respondents (47.4%) reported experiencing depression above the cut-off point of 10, indicating major depression, with moderately severe depression being most frequent (40.6%), followed by severe depression (32.1%) and moderate depression (27.3%). Regarding the impact of depression on daily activities, a majority (48.0%) reported that depression does not make daily activities difficult at all. Some difficulty was experienced by 20.0% of respondents, while 13.9% reported finding daily activities very difficult and 18.1% reported them as extremely difficult. In terms of social support, a majority (53.3%) of respondents reported having at least moderate social support, while a smaller proportion reported low social support (Table 2).

**TABLE 2:** Depression prevalence, severity, impact on daily life and social support among people who inject drugs in Kaduna State (*N* = 525).

Depression	Frequency	Percent
**Depression cut-off point ≥ 10 (major depression)**		
None	276	52.60
Depression	249	47.40
**Depression severity**		
Moderate	68	27.30
Moderately severe	101	40.60
Severe	80	32.10
**Depression-difficulty performing daily activities**		
Not difficult at all	252	48.00
Somewhat difficult	105	20.00
Very difficult	73	13.90
Extremely difficult	95	18.10
**Social support**		
Low	10	1.90
Moderate	280	53.30
High	235	44.80

There is a significant association between age and depression (χ^2^ = 76.5, *p* < 0.001, Phi = 0.382). Individuals aged 25–34 appear to have the highest proportion of depression compared to other age groups. There is also a significant association between sex and depression (χ^2^ = 126.0, *p* < 0.001, Phi = −0.490). Females have a substantially higher proportion of depression compared to males. There is a significant association between marital status and depression (χ^2^ = 115.5, *p* < 0.001, Phi = 0.469).

Individuals who were divorced or single had higher proportions of depression compared to those who were married. The association between employment status and depression was significant (χ^2^ = 24.1, *p* < 0.001, Phi = 0.214). Individuals employed in the private sector had a lower proportion of depression compared to other employment categories (Table 3). The study also found a significant association between education level and depression (χ^2^ = 44.3, *p* < 0.001, Phi = 0.291). Those with tertiary education had a higher proportion of depression compared to those with lower education levels. An association between social support and depression was found to be significant (χ^2^ = 26.8, *p* < 0.001, Phi = 0.226). Individuals with low social support had a lower proportion of depression compared to those with moderate or high social support.

**TABLE 3:** Bivariate analysis of depression and sociodemographic data including social support (*N* = 525).

Variable	Without depression		Depression		Total		Pearson chi-Square test
	*n*	%	*n*	%	*n*	%	
**Age**							
18–24	107	62.2	65	37.8	172	100.0	(χ^2^ = 76.5, *p* < 0.001, Phi = 0.382)
25–34	167	58.4	119	41.6	286	100.0	
35–44	2	4.6	41	95.4	43	100.0	
> 44	0	0.0	24	100.0	24	100.0	
Total	276	61.4	249	38.6	525	100.0	
**Sex**							
Female	16	11.6	122	88.4	138	100.0	(χ^2^ = 126.0, *p* < 0.001, Phi = -0.490)
Male	260	67.2	127	32.8	387	100.0	
Total	276	52.6	249	47.4	525	100.0	
**Marital status**							
Single	235	68.7	106	31.3	342	100.0	(χ^2^ = 115.5, *p* < 0.001, Phi = 0.469)
Married	39	30.5	89	69.5	128	100.0	
Divorced	2	3.6	54	96.4	56	100.0	
Total	276	61.4	249	38.6	525	100.0	
**Employment**							
Student	62	48.1	67	51.9	129	100.0	(χ^2^ = 24.1, *p* < 0.001, Phi = 0.214)
Private sector	118	67.6	57	32.4	175	100.0	
Public sector	49	44.9	60	55.1	109	100.0	
Unemployed	47	42.0	65	58.0	112	100.0	
Total	276	61.4	249	38.6	525	100.0	
**Education**							
Primary	12	60.0	8	40.0	20	100.0	(χ^2^ = 44.3, *p* < 0.001, Phi = 0.291)
Secondary	115	68.9	52	31.1	167	100.0	
Tertiary	128	50.4	126	49.6	254	100.0	
None	21	25.0	63	75.0	84	100.0	
Total	276	61.4	249	38.6	525	100.0	
**Social support**							
Low	4	40.0	6	60.0	10	100.0	(χ^2^ = 26.8, *p* < 0.001, Phi = 0.226)
Moderate	119	42.5	161	57.5	280	100.0	
High	153	65.1	82	34.9	235	100.0	
Total	276	61.4	249	38.6	525	100.0	

This analysis suggests that age, sex, marital status, employment, education and social support were all associated with depression. Younger age, female gender, being divorced or single, unemployment, higher education levels and lower social support all correlated with higher rates of depression.

The analysis revealed significant associations between demographic factors and the likelihood of experiencing depression. Older individuals showed a higher likelihood of depression, with each one-unit increase in age corresponding to a 0.156-unit increase in the log odds of depression (*p* < 0.05). Females exhibited significantly higher odds of experiencing depression compared to males (Exp(B) = 15.684, *p* < 0.001). Divorced individuals had significantly higher odds of depression compared to single individuals (Exp(B) = 9.627, *p* < 0.001), while married individuals also showed higher odds of depression compared to single individuals (Exp(B) = 3.469, *p* < 0.05). Unemployed individuals demonstrated significantly higher odds of experiencing depression compared to those employed in the private sector (Exp(B) = 4.143, *p* < 0.001). Individuals with primary education displayed significantly higher odds of experiencing depression compared to those with no education (Exp(B) = 3.156, *p* < 0.05). Those with moderate social support had significantly higher odds of experiencing depression compared to those with low support (Exp(B) = 2.937, *p* < 0.001).

In summary, based on the logistic regression model, older age, female sex, marital status (particularly being divorced or married), unemployed status and having primary education are significant predictors of depression among the studied population (Table 4).

**TABLE 4:** Binary logistic regression estimates for depression severity.

Variable in the equation	B	s.e.	Sig.	Exp(B)	95% CI for Exp(B)	
					Lower	Upper
**Step 1^a^**						
**Age**	0.156	0.032	0.000	1.168	1.098	1.243
**Sex (1)**	2.753	0.349	0.000	15.684	7.912	31.092
**Marital status**	-	-	0.000	-	-	-
Marital status (1)	2.265	0.818	0.006	9.627	1.938	47.817
Marital status (2)	1.244	0.319	0.000	3.469	1.856	6.483
**Employment status**	-	-	0.000	-	-	-
Employment status (1)	−0.491	0.384	0.201	0.612	0.288	1.299
Employment status (2)	−0.038	0.448	0.933	0.963	0.400	2.319
Employment status (3)	1.421	0.414	0.001	4.143	1.842	9.318
**Level of education**	-	-	0.074	-	-	-
Level of education (1)	1.149	0.453	0.011	3.156	1.300	7.663
Level of education (2)	0.593	0.683	0.385	1.809	0.474	6.904
Level of education (3)	0.147	0.303	0.628	1.158	0.640	2.095
**Social support**	-	-	0.000	-	-	-
Social support (1)	0.529	0.899	0.556	1.697	0.291	9.894
Social support (2)	1.077	0.257	0.000	2.937	1.773	4.864
**Constant**	−6.457	1.013	0.000	0.002	-	-

Note: Step 1a indicates the initial iteration in stepwise logistic regression where significant predictors were selected.

s.e., standard error; CI, confidence interval; Sig., significance; B, regression coefficient.

## Discussion

The purpose of this research was to examine the prevalence of depression and its sociodemographic correlates among 525 PWID. The sample’s preponderance of young individuals is consistent with previous research on trends in substance use, which indicates that young adults frequently engage in riskier behaviour, such as drug injection, at a higher rate.^14^ The sample’s variability in employment status highlights that drug injection behaviours are prevalent across a range of occupational categories. There are disparities in educational attainment within the sample, with a considerable percentage reporting no formal education, suggesting potential barriers to accessing knowledge and resources on safe injection practices. Information on safe injection practices and the adverse effects of substance use is disseminated through various channels, including healthcare facilities, community outreach programmes by non-governmental organisations (NGOs), government initiatives, religious and community leaders, and educational institutions. However, access to these sources may be influenced by educational and socioeconomic factors.

The most commonly injected drug was pentazocine (60.4%). This aligns with the results obtained from research conducted in India.^15,16^ However a national survey conducted in Nigeria in 2009 identified heroin as the most commonly injected drug,^17^ which aligns with findings from studies in Hungary, Addis Ababa and the United States of America.^18,19,20^ In a more recent study, the 2018 United Nations Office on Drugs and Crime survey reported that opioids particularly tramadol, codeine and morphine were the most commonly injected drugs in Nigeria, with a prevalence of 4.7%.^21^ These differences indicate that the most commonly injected substances among individuals who inject drugs differ geographically and over time.

The prevalence of depression in the study population was high despite high levels of social support levels. Prior studies have established that individuals who inject drugs have a higher incidence of depression, or symptoms associated with depression compared to the general population, highlighting marginalised populations’ higher susceptibility to mental health difficulties.^1,3^ Our findings underscore the intricate relationship between sociodemographic variables and depression in PWID. Chi-square analysis revealed significant associations between depression and all six sociodemographic variables, albeit with varying degrees of strength. Subsequent analysis using binary logistic regression provided additional insights into the connection between sociodemographic variables and the severity of depression. Age, sex, marital status, employment status and social support emerged as crucial predictors of depression.

Marital status is consistently identified as a critical determinant of mental health across populations. In our PWID-focussed study, we identified a higher prevalence of depression among divorced and married persons. This is consistent with Niroula’s study conducted in 2020 on depressive disorder patients and Islam’s population-based study conducted in 2017,^22,23^ which found that being married is associated with an increased risk of depression. Niroula’s study highlights how marital dynamics can contribute to the onset of depressive symptoms, particularly emphasising the psychological stress associated with marital dissatisfaction. The Reddon study in 2018, and Levintow’s 2022 research on HIV-positive men who inject drugs in Vietnam underscore the importance of marital status by establishing that single status is a significant predictor of severe depressive symptoms.^1,24^ Reddon’s study is particularly notable for its focus on the intersection of HIV and substance use, underscoring how single status may increase the psychological burden experienced by individuals because of a lack of social support. This is consistent with our research outcomes, which indicate that marital status significantly influences the mental health of substance use abusers. Islam’s study provides a broader perspective, correlating marital status with the severity of major depressive disorder.^23^ The positive correlation between marital status and severity in their findings echoes the trend observed in our study. This finding implies that marital status has an impact not only on the likelihood of developing depression but also on the severity of depressive symptoms in individuals who inject drugs.

Age-related patterns vary considerably between populations. In our study on PWID, older age groups, particularly those over 44, had higher depression rates. In contrast, Iliev’s 2020 research on psychiatric patients found a higher frequency among men aged 25 to 34 years.^25^ Iliev’s study is notable for its focus on a specific age range within a psychiatric setting, revealing that younger persons in this demographic may have higher rates of depression because of factors such as developmental stressors and transitional life stages. Hays’ 2017 study of risk typology in African Americans adds an intriguing dimension by categorising age groups within risk patterns.^26^ This shows that a variety of protective and risk factors could mediate the link between age and depression. The high protective or low-risk type, distinguished by elevated social support and religiosity levels, may suggest that specific age cohorts derive greater advantages from protective elements. Understanding these discrepancies underscores the necessity for interventions that are customised to address the distinct obstacles encountered by various demographic groups.

Gender disparities exist consistent with previous studies on injection drug users. Our research on PWID shows that females have a higher risk (15.6 odds) of having depression compared to males in other studies.^1,24,27^ Addressing and understanding gender-specific risk factors remain critical in comprehensive mental health programmes, as is designing interventions that address the particular needs of both male and female PWID. Hays’ study of risk typology in African Americans offers a complex approach, implying that gender may interact with other characteristics, such as religiosity and social support, to affect the risk of depression.^26^ This supports the notion that taking into account a variety of protective and risk factors is necessary to comprehend the gender-specific features of mental health.

Educational status exhibits varying associations with depression. Both our study and Jinghua Li’s research in China show a relationship between those with lower education and a higher risk of depression.^28^ According to Li’s study, lower education levels correlate with higher rates of depression, which may be attributed to limited access to resources and increased stressors associated with lower educational backgrounds. However, the specific patterns vary, emphasising the need to consider regional and cultural variables when examining the association between education and mental health among PWID. Iliev’s epidemiological study in 2020 on major depression among hospitalised patients supports the existing findings, highlighting a higher prevalence among those with lower educational attainment suggesting that lower educational attainment may contribute to the development of depression through mechanisms such as reduced socioeconomic opportunities and increased life stress.^25^ Similarly, Islam’s population-based study conducted in 2017 provides additional insight by examining the severity of major depressive disorder in relation to educational status.^23^ The study found that poorer education is connected with a higher severity of major depressive disorder. These observed patterns highlight the universality of the correlation between education and depression among different populations.

Employment dynamics play a role in mental health outcomes. The present study’s results are consistent with those of Reddon’s research conducted in 2018,^24^ which examined the prevalence of mental health disorders among individuals who inject drugs and found a positive correlation between unemployment and depression diagnosis. The complexities of work status and its impact on mental health are further highlighted by Levintow’s 2022 study.^1^ The study focusses on the interplay between employment status, social support and mental health, highlighting that unemployment often exacerbates psychological distress because of the lack of financial stability and social support networks.

The logistic regression of the present study demonstrates the association between being unemployed and an increased risk of depression. This finding aligns with existing research trends linking unemployment to depression. Prior research across diverse populations, not exclusive to injection drug users, associates job insecurity with psychological distress.^29^ The non-significant effects of being employed in either the private or the public sector in this study may be influenced by factors such as social support, healthcare access or coping mechanisms. Further exploration is needed to understand the nuanced relationship between unemployment status and depression among PWID.

Lastly, our study examined the influence of social support on depression risk. Surprisingly, individuals with a moderate level of support demonstrated higher odds (2.9) of depression compared to those with low support. This unexpected finding may indicate that individuals with moderate support may experience heightened expectations or interpersonal conflicts, leading to psychological distress. Further research is needed to explore the nuanced dynamics of social support and its impact on mental health outcomes. Our findings are inconsistent with Hays’ study on African Americans and Risser’s research on injection drug users regarding the protective role of social support against depression and depressive symptoms.^26,27^ Both pieces of research underline the role of social relationships in reducing depression symptoms. Promoting social support continues to be a recurring element in efficacious interventions for mental health. In a Canadian study, social support served as a protective factor against non-fatal drug overdose among PWID.^5^ Similarly, a study conducted in Kazakhstan, Central Asia, while not directly examining the social support–depression relationship, shed light on the prevalence of depression among PWID and their intimate partners.^4^ These disparate results underscore the complex nature of social support’s impact on mental health outcomes, influenced by cultural norms and contextual factors. Further research is needed to clarify these relationships and guide targeted interventions for PWID. Recognising the ongoing relevance of social support, therapies for PWID should target efforts that strengthen community and familial relationships. Niroula’s research findings, which suggest that rural regions have a higher prevalence of depression, stimulate contemplation regarding the potential regional variations in social support.^20,21^ The presence of unique social dynamics in rural areas can influence the accessibility and characteristics of support networks. This raises concerns regarding the quality and accessibility of social services in various circumstances.

The variability in outcomes from studies on PWID highlights the varied character of sociodemographic patterns in depression. Although some trends are not limited to specific populations, the variations highlight the importance of tailoring interventions to the context. Adapting mental health interventions to the distinct sociodemographic characteristics of various populations promotes enhanced and focussed strategies, which ultimately contribute to the enhancement of mental health in diverse communities.

## Conclusion

### Limitations and future directions

Although our research provides valuable contributions, it is important to acknowledge certain limitations. Our ability to establish causation is limited by the cross-sectional design of the data. The reliance on self-reported data posed a challenge, hindering independent verification of respondent-provided information. The use of a screening instrument rather than a standardised diagnostic tool may have resulted in inaccurate estimates of depression prevalence and severity, limiting the accuracy of mental health assessment in this study. Despite efforts to mitigate social desirability bias by ensuring respondent anonymity, its influence on participants’ responses remains possible. An absence of identifiers in the study raises concerns about follow-up and cross-referencing responses. Additionally, recruitment challenges—where 44% of individuals approached declined participation during the consent process—may have led to selection bias, potentially affecting the representativeness of the sample. These limitations, recognised by the study, should be considered when interpreting findings, and recognising potential impacts on data completeness, accuracy and generalisability.

In conclusion, our research sheds light on the intricate relationships between sociodemographic factors and depression among PWID. Understanding these dynamics is critical for creating effective, focussed interventions to meet the unique mental health requirements of this population. The multidimensional nature of these relationships underscores the importance of holistic and context-specific approaches to mental healthcare and preventative initiatives for depression in PWID.

## References

[1] Levintow SN, Pence BW, Sripaipan T, et al. The role of depression in secondary HIV transmission among people who inject drugs in Vietnam: A mathematical modelling analysis. PLoS One. 2022;17(10):e0275995. 10.1371/journal.pone.0275995 36240142 PMC9565425

[2] Reyes J, Robles R, Colón H, et al. Severe anxiety symptomatology and HIV risk behavior among Hispanic injection drug users in Puerto Rico. AIDS Behav. 2007;11:145–150. 10.1007/s10461-006-9090-x 17122902

[3] Colledge S, Larney S, Peacock A, et al. Depression, post-traumatic stress disorder, suicidality and self-harm among people who inject drugs: A systematic review and meta-analysis. Drug Alcohol Depend. 2020;207:107793. 10.1016/j.drugalcdep.2019.107793 31874449

[4] Shaw SA, El-Bassel N, Gilbert L, et al. Depression among people who inject drugs and their intimate partners in Kazakhstan. Community Ment Health J. 2016;52(8):1047–1056. 10.1007/s10597-015-9883-3 25963238 PMC4643466

[5] Pabayo R, Alcantara C, Kawachi I, Wood E, Kerr T. The role of depression and social support in non-fatal drug overdose among a cohort of injection drug users in a Canadian setting. Drug Alcohol Depend. 2013;132(3):603–609. 10.1016/j.drugalcdep.2013.04.007 23647731 PMC3770831

[6] Ali OJ, Felix SO, Sheila O, Monday O, Awelewa O. Geographical disparities of people who inject drugs and associated needle sharing in the selected states in Nigeria: A call for urgent intervention programmes. Niger J Health Sci. 2022;22:11–16. 10.4103/njhs.njhs_7_22

[7] Bruckner TA, Scheffler RM, Shen G, et al. The mental health workforce gap in low- and middle-income countries: A needs-based approach. Bull World Health Organ. 2010;89:184–194. 10.2471/BLT.10.082784 21379414 PMC3044251

[8] Wang PS, Aguilar-Gaxiola S, Alonso J, et al. Use of mental health services for anxiety, mood, and substance disorders in 17 countries in the WHO world mental health surveys. Lancet. 2007;370:841–850. 10.1016/S0140-6736(07)61414-7 17826169 PMC2847360

[9] Patel V, Maj M, Flisher AJ, et al. Reducing the treatment gap for mental disorders: A WPA survey. World Psychiatry. 2010;9(3):169–176. 20975864 10.1002/j.2051-5545.2010.tb00305.x PMC2953637

[10] Buckingham E, Schrage E, Cournos F. Why the treatment of mental disorders is an important component of HIV prevention among people who inject drugs. Adv Prev Med. 2013;2013(1):690386. 10.1155/2013/690386 23401785 PMC3562640

[11] Kaduna State Bureau for Statistics. Demography [homepage on the Internet]. Kaduna State Bureau for Statistics. [cited 2024 Aug 26]. Available from: https://kdbs.ng/domains/demography/?publication=

[12] Karon JM, Wejnert C. Statistical methods for the analysis of time-location sampling data. J Urban Health. 2012;89:565–586. 10.1007/s11524-012-9676-8 22421885 PMC3368048

[13] Adewuya AO, Ola BA, Afolabi OO. Validity of the patient health questionnaire (PHQ-9) as a screening tool for depression amongst Nigerian university students. J Affect Disord. 2006;96(1–2):89–93. 10.1016/j.jad.2006.05.021 16857265

[14] Ritchwood TD, Ford H, DeCoster J, Sutton M, Lochman JE. Risky sexual behavior and substance use among adolescents: A meta-analysis. Child Youth Serv Rev. 2015;52:74–88. 10.1016/j.childyouth.2015.03.005 25825550 PMC4375751

[15] Avvaru K, Pasagadugula K, Rajsekhar K, Kumar S. Behavioral patterns of injectable drug users (IDUs) in Visakhapatnam City. IOSR J Dent Med Sci. 2014;13:45–47. 10.9790/0853-13714547

[16] Ambekar A, Rao R, Mishra A, Agrawal A. Type of opioids injected: Does it matter? Drug Alcohol Rev. 2015;34(1):97–104. 10.1111/dar.12208 25302827

[17] Adamson TA, Ogunlesi AO, Morakinyo O, et al. Descriptive national survey of substance use in Nigeria. J Addict Res Ther. 2015;6:234. 10.4172/2155-6105.1000234

[18] Gyarmathy V, Péterfi A, Figeczki T, et al. Diverted medications and new psychoactive substances: A chemical network analysis of discarded injecting paraphernalia in Hungary. Int J Drug Policy. 2017;46:61–65. 10.1016/j.drugpo.2017.05.003 28628897

[19] Deyessa N, Senbete B, Abdo A, Mundia B. Population estimation and harm reduction among people who inject drugs in Addis Ababa, Ethiopia. Harm Reduct J. 2020;17(1):61. 10.1186/s12954-020-00407-x 32894153 PMC7487880

[20] Biancarelli D, Biello K, Childs E, et al. Strategies used by people who inject drugs to avoid stigma in healthcare settings. Drug Alcohol Depend. 2019;198:80–86. 10.1016/j.drugalcdep.2019.01.037 30884432 PMC6521691

[21] United Nations Office on Drugs and Crime (UNODC). Drug use in Nigeria 2018 [homepage on the Internet]. Vienna: UNODC; 2019 [cited 2024 Aug 19]. Available from: https://www.unodc.org/documents/data-and-analysis/statistics/Drugs/Drug_Use_Survey_Nigeria_2019_BOOK.pdf

[22] Niroula R, Upadhyay HP. Socio-demographic profile of patients suffering from depressive disorder attending psychiatry outpatient department. J Chitwan Med Coll. 2020;10(2):10–13. 10.3126/jcmc.v10i2.29661

[23] Islam MR, Adnan R. Socio-demographic factors and their correlation with the severity of major depressive disorder: A population-based study. World J Neurosci. 2017;7(2):193. 10.4236/wjns.2017.72014

[24] Reddon H, Pettes T, Wood E, et al. Incidence and predictors of mental health disorder diagnoses among people who inject drugs in a Canadian setting. Drug Alcohol Rev. 2018;37(S1):S285–S293. 10.1111/dar.12631 29168263 PMC5940561

[25] Iliev B, Bonevski D, Naumovska A. Epidemiological characteristics of major depression of hospitalized patients in psychiatric hospital ‘Demir Hisar’ – Demir Hisar for Five years from 2013 to 2017. Open Access Maced J Med Sci. 2020;8(A):378–384. 10.3889/oamjms.2020.3555

[26] Hays K. A risk typology for depression in African Americans. Issues Ment Health Nurs. 2017;38(10):812–821. 10.1080/01612840.2017.1342727 28745919

[27] Risser J, Cates A, Rehman H, Risser W. Gender differences in social support and depression among injection drug users in Houston, Texas. Am J Drug Alcohol Abuse. 2010;36(1):18–24. 10.3109/00952990903544802 20141392

[28] Li J, Gu J, Lau J, et al. Prevalence of depressive symptoms and associated factors among people who inject drugs in China. Drug Alcohol Depend. 2015;151:228–235. 10.1016/j.drugalcdep.2015.03.028 25920800

[29] Kim TJ, Von dem Knesebeck O. Perceived job insecurity, unemployment and depressive symptoms: A systematic review and meta-analysis of prospective observational studies. Int Arch Occup Environ Health. 2016;89:561–573. 10.1007/s00420-015-1107-1 26715495

